# An evaluation of the error and uncertainty in epibenthos cover estimates from AUV images collected with an efficient, spatially-balanced design

**DOI:** 10.1371/journal.pone.0203827

**Published:** 2018-09-18

**Authors:** Jacquomo Monk, Neville S. Barrett, David Peel, Emma Lawrence, Nicole A. Hill, Vanessa Lucieer, Keith R. Hayes

**Affiliations:** 1 Institute for Marine and Antarctic Studies, University of Tasmania, Hobart, Tasmania, Australia; 2 Data61, Commonwealth Scientific and Industrial Research Organization (CSIRO), Hobart, Tasmania, Australia; 3 Data61, Commonwealth Scientific and Industrial Research Organization (CSIRO), Dutton Park, Queensland, Australia; Department of Agriculture and Water Resources, AUSTRALIA

## Abstract

Efficient monitoring of organisms is at the foundation of protected area and biodiversity management. Such monitoring programs are based on a systematically selected set of survey locations that, while able to track trends at those locations through time, lack inference for the overall region being “monitored”. Advances in spatially-balanced sampling approaches offer alternatives but remain largely untested in marine ecosystems. This study evaluated the merit of using a two-stage, spatially-balanced survey framework, in conjunction with generalized additive models, to estimate epifauna cover at a reef-wide scale for mesophotic reefs within a large, cross-shelf marine park. Imagery acquired by an autonomous underwater vehicle was classified using a hierarchical scheme developed under the Collaborative and Automated Tools for Analysis of Marine Imagery (CATAMI). At a realistic image subsampling intensity, the two-stage, spatially-balanced framework provided accurate and precise estimates of reef-wide cover for a select number of epifaunal classes at the coarsest CATAMI levels, in particular bryozoan and porifera classes. However, at finer hierarchical levels, accuracy and/or precision of cover estimates declined, primarily because of the natural rarity of even the most common of these classes/morphospecies. Ranked predictor importance suggested that bathymetry, backscatter and derivative terrain variables calculated at their smallest analysis window scales (i.e. 81 m^2^) were generally the most important variables in the modeling of reef-wide cover. This study makes an important step in identifying the constraints and limitations that can be identified through a robust statistical approach to design and analysis. The two-stage, spatially-balanced framework has great potential for effective quantification of epifaunal cover in cross-shelf mesophotic reefs. However, greater image subsampling intensity than traditionally applied is required to ensure adequate observations for finer-level CATAMI classes and associated morphospecies.

## Introduction

Research programs worldwide aim to monitor biodiversity in an effort to identify conservation priorities and assess management actions [1]. Monitoring for conservation can be challenging because adequately estimating abundance, occurrence, cover or species richness can be hampered by logistical problems associated with access to the region of interest [2] and accurate detection of species [3, 4]. As a result, many researchers are trying to identify efficient surveying tools that will quantitatively assess the status and distribution of species, often with varying life history and habitat requirements.

Despite this increased interest and investment in monitoring programs, resources are limited, often resulting in the need to undertake multiple objectives simultaneously [5]. Ideally, an efficient survey design would be tailored to a specific, narrow set of objectives (e.g., monitoring the recovery of a species). However, over the longer term, the focus of monitoring programs may change as more is learned about the ecosystem(s) being monitored or as management questions/priorities evolve. In such instances, a survey design highly tailored to a specific set of objectives will become obsolete. Ultimately, we need to strategically choose which, of the many components of an ecosystem should be monitored, survey locations to target, and the survey tool to collect the monitoring data in an attempt to maximize conservation outcomes.

In addition to survey designs being flexible, monitoring sites need to be representative of the area or population of interest. Increasingly, monitoring programs are moving away from *a priori* choosing specific and potentially unrepresentative sites to monitor (judgemental sampling) to probabilistic sampling where every part of the survey area or population has some chance of being surveyed. Spatially-balanced probabilistic survey designs, which as the name suggests spreads samples well throughout the survey area, are considered state-of-art (e.g., [6, 7–10]) because they are efficient and flexible. It is only recently that spatially-balanced designs have been applied in the assessment of marine ecosystems, including demersal and pelagic marine fishes [8, 11, 12] and seafloor habitats [13]. They are being considered as the standard for designing/conducting multiple-objective monitoring within Australia’s Marine Park (AMP) network and here we examine their utility for quantitatively estimating seafloor epifaunal communities. Typically, shelf habitats found in AMPs are beyond diving depths (below 30 m), so are unable to be surveyed using traditional scuba-based approaches commonly used in biodiversity-based monitoring programs (e.g., [14]). Until recently, these deeper shelf habitats had rarely been quantitatively surveyed for the cover of epifaunal communities due to the absence of appropriate tools. Technological developments in sophisticated tools over the last decade mean that it is now possible to photographically survey the epifaunal communities associated with the seabed using geo-located stereo photography with high degrees of positional accuracy [15, 16]. Autonomous underwater vehicles (AUVs) are at the forefront of such tools, and can capture precisely geo-located images along pre-programed transects, and at a consistent elevation above the seabed to maintain a constant search area for subsequent scoring of imagery [15, 17].

Traditionally such imagery is semi-qualitatively scored in a top-down approach to identify broad habitats or biotopes for mapping purposes (e.g., the European Nature Information System [18]). Increasingly, more detailed information than habitat or biotope type is required from such imagery. However, precise, species level, taxonomic identification (such those achieved in the World Register of Marine Species [19]) from imagery is often difficult without physical sampling of specimens and exhaustive species catalogues to provide validation [20]. To address the need for standard approaches for identifying biota from imagery, Althaus et al. [21] developed a hierarchical classification scheme for scoring biological components observed in such imagery, allowing imagery to be scored from multiple levels from Phyla to morphospecies. Known as the Collaborative and Automated Tools for Analysis of Marine Imagery (CATAMI), the scheme enables the hierarchical classification of imagery beyond broad habitat or biotope.

In this research paper we investigate the outcomes of when such survey tools and classification schemes are coupled with spatially-balanced sampling designs. We use the spatially-balanced sampling design applied to AUV transects and images to calculate estimates of epifaunal cover and the associated uncertainties at multiple levels of the CATAMI biological hierarchy. We aim to assess the development of a method for obtaining accurate baseline estimates of epifaunal cover at whole-of-reef scales. The outcome, if successful, will be used to inform the quantitative inventory, monitoring and management of biodiversity on shelf reef systems within the network of AMPs. The validation of this survey design represents an important step in the adoption of AUV-based monitoring of AMP network.

## Materials and methods

### Study site

**Fig 1:**
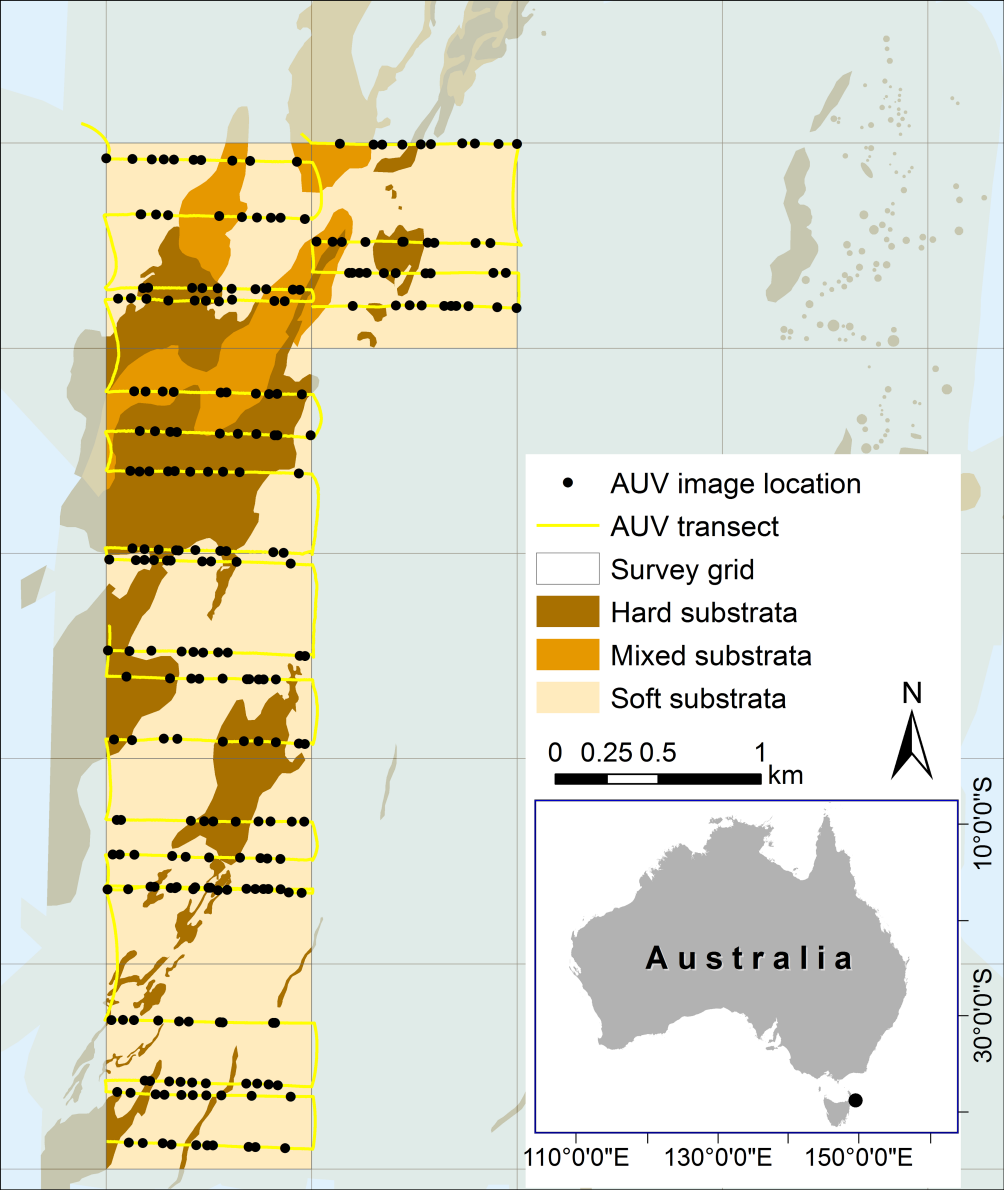
Study area map showing locations of autonomous underwater vehicle transects and images selected for interrogation of epibenthos cover. Underlying data show the substrata classification across the broader region that was produced by Lawrence et al. [15]. Boxes show windows used to constrain sampling extent where a considerable proportion of reef habitat was mapped.

The study was located in the multiple use zone (IUCN VI) of the Flinders Marine Park (40°37’S, 148°46’ E). The Flinders Marine Park is approximately 20 km offshore of northeastern Tasmania, Australia (Fig 1), and is influenced by southward incursions of the East Australian Current (EAC) in summer months. As a result, the biota of the region includes a mixture of cold-temperate water species, as well as organisms more commonly found in warmer temperate waters [22, 23]. The seafloor is dominated by soft sediments with isolated patches of low profile reef [24], that are characterized by slightly dipping sedimentary rock formations that erode the bedding planes to make long, linear ledge features of 1–2 m in height [25]. These reefs support a variety of sessile invertebrates including porifera, hydrozoans, bryozoans and ascidians that are thought to be characteristic of the broader region of eastern Tasmania [25–27]. We selected a large isolated reef at the continental shelf edge as the basis of our study to describe faunal cover at whole-of-reef scales.

Appropriate ethics (University of Tasmania Animal Ethics Permit: A12514) and fieldwork (Australian Government Director of National Parks Approval of Research Activities in the Southeast Commonwealth Marine Park Network: Ref. No 07/10622) permits were obtained for this work.

### Multibeam sonar data

The multibeam sonar (MBS) data were acquired using a hull-mounted Kongsberg EM3002 multibeam sonar on a 22-m research vessel, with the data being logged in Kongsberg acquisition software. Post-processing was completed in Caris HIPS and SIPS software to remove artefacts. The final bathymetric and backscatter intensity outputs were processed at 3 m horizontal resolution for subsequent analysis in a GIS platform.

From the bathymetric surface, seven seabed terrain variables were derived: eastness, northness, structural complexity (i.e. slope of the slope; S1 Fig), rugosity, slope, plan curvature, profile curvature and maximum curvature (S1 Table). The derived variables were calculated in ArcGIS spatial analyst or LandSerf [28] in order to quantify a range of structural attributes across the Flinders Marine Park study area (S1 Table). Additional variables of latitude and longitude were also generated for the study area. The bathymetrically-derived variables were calculated at five analysis window scales of 3x3, 9x9, 17x17, 33x33 and 65x65 cells, equating to on-ground window sizes of 81, 729, 2601, 9,801, and 38025 m^2^ (as in [29]). Multiple spatial scales were considered because physical and biological processes, such as exposure to current circulation, food particle delivery, and species interactions, operate on various spatial scales [29–33]. Thus, including them is known to improve model accuracies [34].

### AUV imagery

Seabed images were collected with a synchronized pair of high sensitivity 12 bit, 1.4 megapixel cameras (AVT Prosilica GC1380 and GC1380C; one monochrome and one color) fitted to a modified Seabed class AUV, detailed in Williams et al. [15]. The position of the AUV was calculated using a doppler velocity log including a compass with integrated roll and pitch sensors and ultra-short baseline acoustic positioning system [15].

We were primarily interested in reef habitat within this region, so we limited the survey extent to the area that contained hard-substratum identified from the classification of MBS data collected in this region [13]. A 1x1 km grid was overlaid on the area containing hard-substratum and the starting point of transects selected (using the spatially-balanced approach known as generalize random tessellation stratified; GRTS [6]) within each 1 km grid with equal inclusion probability. In the field we were able to complete 24 spatially-balanced transects with four transects in each of six grid cells. These AUV transects covered depths of *c* 60–92 m. Each AUV transect was pre-programmed so that the AUV tracked the seabed at an altitude of ~ 2 m at a cruising speed of 0.5 ms^-1^, capturing an image every 0.5 s with an approximate width of the field of view of 1.5–2.5 m per image. All surveys were conducted during daylight hours over three days in June 2013.

Scoring of imagery from the AUV was undertaken using TransectMeasure software (www.seagis.com). The objective of this research was to determine if reliable estimates of cover could be achieved at the finest possible taxonomic resolution, factoring in the time it takes to score AUV imagery. Ten images selected using GRTS along a line (generalized random interval sampling) were scored using 50 random points superimposed on each image and the underlying biota classified to morphospecies (i.e. “species” were distinguished based on morphological differences such as shape and/or color). Morphospecies to the size of at least 2 cm could be reliably differentiated. Each morphospecies was assigned a parental hierarchical CATAMI class, which enable morphospecies to be grouped hierarchically to determine the best taxonomic resolution for estimates of cover. The CATAMI scheme has up to six levels, ranging from broad taxonomic classes (level 1) to a reasonably fine level (level 6) that combines taxonomy and physical morphology [21].

### Analytical approach

The selection of AUV images described above is a two-stage, spatial-balanced sampling design based on GRTS where the start points of the transects and the selected images within those transects are randomized. It is important to note that we have not applied GRTS in the original manner where spatial balance is usually achieved by randomizing in two dimensions simultaneously because efficiency would be severely reduced if the AUV were to be regularly moved from site to site. Accordingly, traditional GRTS estimators do not apply. The selection of images within the transect using 2^nd^ stage of spatial balance was chosen as it decreased spatial-autocorrelation between samples when compared to simple random designs with mean Moran’s I values at CATAMI level 1 of 0.08±0.02SE and 0.03±0.02SE for random and spatially-balanced samples, respectively.

We used generalized additive models (GAMs), which are flexible, nonparametric generalizations of generalized linear regression [35], to provide estimates of area-wide epifaunal cover based on the scored AUV imagery and the multiscale seabed variables. The GAMs are used as a model-based approach to calculate estimates that can accommodate various sampling/design regimes if sampling is reasonably representative of the covariate space, and there are sufficient data to estimate model parameters, which are intrinsic properties of a well-designed, spatially-balanced sample. Using a combination of probabilistically sampled data and model-based analysis (GAMs) provides some protection against model miss-specification whilst allowing the calculation of estimates across a fine-scale grid (see [36]).

An individual binomial GAM was constructed for each hierarchical CATAMI class using *‘mgcv’* package in R software. The GAMs were fitted with a cubic spline smooth and four degrees of freedom after varying these parameters [37]. The models were constructed manually in a backward stepwise manner. Terms were removed from the model such that each step resulted in the smallest significant reduction in residual deviance when compared to the previous model using an approximate chi-square test [37]. Only significant terms were retained in each GAM. Explained deviance (d^2^) of each model was also compared as a measure of accuracy, taking into account the number of degrees of freedom [38]. Cover estimates were calculated by approximately integrating the GAM across the region. That is, predicting from the GAM across a fine-scale grid of equally spaced points and taking the sum. The coefficient of variation (C.V.) was used as a measure of precision for these cover estimates, and was calculated based on the variance approximated using the delta method (see [39]). It represents the variance of the cover estimated by the model, based on the variance of the model predictions [37]. What constitutes an acceptable level of accuracy and precision will vary depending on the context and specifically the inference questions being asked. Here, we considered any model with a d^2^ > 0.6 as adequately accurate [40] and C.V. values < 0.3 as adequately precise [41].

Covariates were standardized prior to inclusion in GAMs so that variable importance could be calculated. Ranked variable importance was calculated by counting the number of times (as a proportion), that particular variable was retained in the final GAM for each CATAMI class or morphospecies.

## Results

### Description of epifaunal assemblage

A total of 127 morphospecies were identified in the AUV imagery (S2 Table). Of these, only 16% were observed greater than 5 times, with 50% being singletons (i.e. only recorded once). Representatives from the porifera CATAMI class dominated the assemblage with 88 morphospecies being identified, followed by 14 morphospecies from the cnidarian CATAMI class, and eight morphospecies each of ascidian and bryozoan classes (S2 Table). Within the porifera CATAMI class, massive, branching and encrusting forms were most common.

### Model-based estimates of epifaunal cover

Of the 127 morphospecies recorded, only 19 were observed frequently enough (i.e. > 5 observations) to derive model-based estimates of cover (S2 Table). These 19 morphospecies consisted of 14 porifera, two cnidarian, and two bryozoan morphospecies, as well as a mixed undistinguishable cnidarian/bryozoan/hydroid matrix class (S1 and S2 Tables). Accuracy, as described by d^2^, for these 19 morphospecies varied considerably from a low 4.0 to a respectable 67.6%, with a mean of 25.3% (S2 Table). Precision (i.e. C.V.) also varied widely from 0.06 to 0.84 (S2 Table). Subsequent estimates of cover were quite low, with values ranging from 0.005 to 5.3% of the study region, reflecting the low prevalence of component epifauna within the study site (S2 Table).

Out of the 19 morphospecies modeled, only 21% (i.e. 4 classes) exhibited precision values < 0.3 (Table 1), with none of these also yielding accuracies > 60% (i.e. accurate and precise cover estimates; Table 1). There also appeared to be no clear pattern between accuracy and precision, meaning that accurate estimates could yield low precision values, and vice versa (S2 Table). For example, the morphospecies with the highest accuracy (i.e. “bryozoan 3 *Cantinicella* like” with a d^2^ = 67.6) had low precision (C.V. = 0.57; S2 Table). By contrast, the morphospecies “simple erect 1 cream” had low accuracy (i.e. d^2^ = 24.9), yet high precision (C.V. = 0.10; S2 Table).

**Table 1:** Summary of model precision and accuracy for epifaunal classes recorded in each taxonomic hierarchy. Complete model outputs are provided in S2 Table.

CATAMI level	No. of classes recorded	Proportion of classes modeled	No. met accuracy criteria (d^2^ > 0.6)	No. met precision criteria (C.V. < 0.3)	Proportion met both criteria
1	7	0.71	5	3	0.29
2	15	0.60	9	2	0.07
3	20	0.60	12	2	0
4	6	0.83	5	0	0
5	4	0.50	2	0	0
6	3	0.67	2	0	0
Morphospecies	127	0.15	19	1	0

Of note was the fact that morphospecies had to be grouped to their broadest parental CATAMI class (i.e. CATAMI levels 1 or 2) before accurate and precise estimates of epifaunal cover were achieved (Table 1 & S2 Table). However, even at these broad CATAMI hierarchies there were only three CATAMI classes that met the accuracy and precision criteria, including bryozoan class (at CATAMI level 1), bryozoan (soft) class (at CATAMI level 2) and porifera (CATAMI level 1) (Table 1 & S2 Table).

### Ranked predictor importance

**Fig 2:**
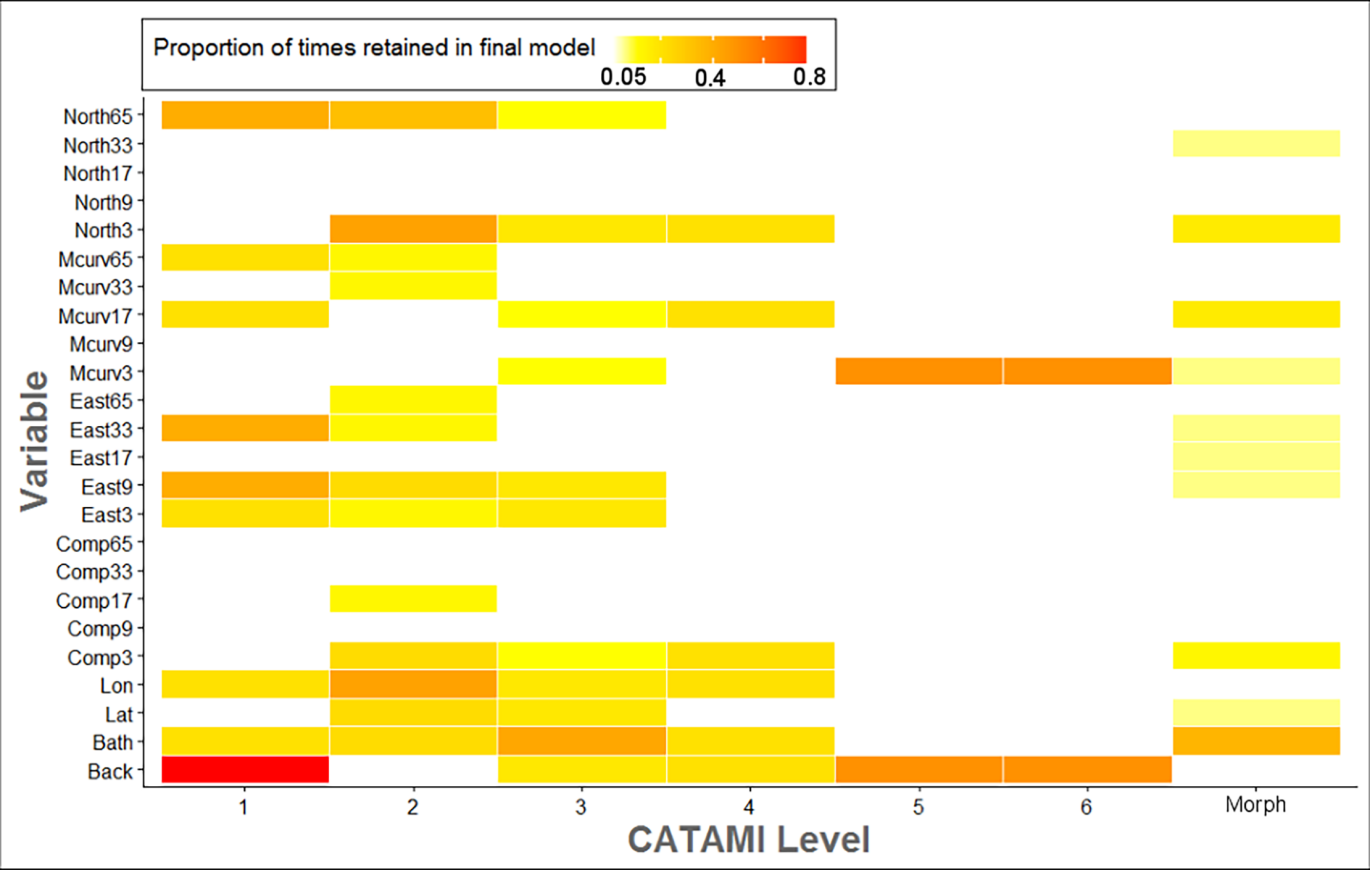
Summary of ranked predictor importance based on predictor variables retained in final models. White cells indicate variable was never retained in final model for any of the classes or morphospecies. Full names are described in S1 Table.

The number of predictors retained in the final models varied between CATAMI classes and morphospecies, and across taxonomic hierarchies (Fig 2). Overall, bathymetry and backscatter intensity were the top-ranked variables, being retained in at least one model for five of the seven taxonomic hierarchies (Fig 2). At the broadest class (i.e. CATAMI level 1) backscatter was the most important variable, being retained in ~80% of the models (Fig 2). Interestingly, however, backscatter was not retained in any of the models at the morphospecies level (Fig 2).

Generally, variables at the smallest analysis window scale (i.e. 3x3 cells) were retained more frequently in final models than their broad-scale counterparts. For example, complexity was retained in four of the seven taxonomic levels at the 3x3 cell analysis window scale, while its broader scale counterparts were almost never retained in the final models, with the exception of one model at CATAMI level 2, which retained complexity at a 9x9 cell analysis window scale (Fig 2).

## Discussion

It is important to conduct a critical evaluation of a survey tool and sampling design prior to it being integrated into an ongoing monitoring program. This is largely because if data from such an approach do not result in sufficient accuracy and precision, then inferences used for management of the target ecosystem(s) may be poor or even incorrect [42]. Here, we undertook an assessment of baseline inventory, which may be employed for a future monitoring program whilst simultaneously evaluating a two-stage, sampling design to quantitatively estimate the cover of reef-associated morphospecies and classes at the whole-of-reef scale based on transect subsamples. We found that greater image subsampling intensity than applied here would be required to provide accurate and precise estimates of reef-wide cover of finer-level CATAMI classes and associated morphospecies.

It is unsurprising that only three broad-level CATAMI classes could be modeled with sufficient accuracy and precision because nearly 98% of morphospecies and 67% of parental CATAMI classes were observed in 10 or fewer AUV images (S2 Table). The link between sample size and model accuracy is well established in the literature, with studies suggesting that somewhere between 30 to 100 observations are sufficient to generate robust models of species distribution (e.g., [43, 44–46]). Here, we could not successfully meet our accuracy and precision criteria until we had observations of 250 or more to fit our models. This indicates that substantially larger sample sizes than suggested by previous research may be required to achieve accurate and precise estimates of epifaunal cover using occurrence datasets from AUV imagery. It is therefore recommended that a minimum of 250 observation records are used with this approach. However, attaining such a sample size is clearly difficult because seabed assemblages around Australia are well known to be dominated by as many as 30–80% singletons (e.g., [25, 47, 48]). Accordingly, no single survey design or sampling tool can be expected to capture the abundance/cover of all organisms with high degrees of accuracy and precision. It is therefore important to consider ways to increase sample sizes and thus potentially increase accuracy and precision of estimates from models.

A simple consideration for achieving improved accuracy and greater precision may be to increase the number of images scored per transect and/or the number of points scored within each image. Studies have shown that a large number of points per image (up to 100) may be needed to adequately capture the diversity of organisms within an image (e.g., [49]). Importantly, however, Van Rein et al. [50] and Perkins et al. [51] suggest that, while a higher number of points per image can increase the detection rate of more organisms within an image, increasing the number of scored images using fewer points is likely to have a similar effect. Ideally, increasing both the number of images scored and the number of points scored within an image would result in greater power to improve the accuracy and precision of epifaunal cover estimates [52]. Unfortunately, the adoption of this approach is likely to result in substantial increases in processing time and therefore cost. Alternatively, targeted scoring could be used, whereby each image is scored for a select number of key indicator organisms. However, the selection of indicator organisms is somewhat difficult, and an often subjective exercise, especially where there is little or no existing biological information available for the ecosystem being studied [53].

Overall, we advocate the use of a multi-tiered scoring approach, whereby a master sample is created which provides a list of images that are spatially balanced provided that they are scored in order. An initial subset of these master sample images is then scored, and organisms recorded from this subset of images are then cross-tabulated to establish a list of numerically abundant organisms for the study ecosystem. This list could then be used to facilitate targeted scoring across a subset of the remaining images in the master sample to strengthen the sample size of these key organisms/classes. Alternatively, if the whole assemblage is important to the study (i.e. an inventory of the organisms’ present) then, instead of targeted scoring the second subset of the spatially-balanced master sample images, these images could be scored the same way as the initial subset, and the process repeated until sufficient sample sizes are obtained. By using either approach, greater power will be achieved, resulting in increased accuracy and precision of epifaunal cover estimates, whilst mitigating the additional time and money associated with unnecessary scoring of additional images.

Another consideration that is likely to improve the precision and accuracy of models is the choice of predictor variables used to extrapolate cover estimates across space. The ranked importance revealed that, in addition to backscatter and bathymetry, fine-scale predictor variables were often more important than their broad-scale counterparts. This is perhaps unsurprising given previous research in the region has highlighted the importance of fine-scale reef ledge features in driving patterns in sessile epifaunal [25]. While we used a multiscale approach to derive predictor variables, as it is widely regarded to be superior to single scale models [29, 30, 32], we were limited to spatial- and MBS-derived predictor variables. Other physical factors influence the distribution and abundance of seafloor biota. For example, variables describing wave/current exposure at the seabed are known to improve predictions of seabed associated flora and fauna (e.g., [54, 55, 56]). Biological productivity and chemical variables are other variables that have been used explain the distribution of cold-water corals, however usually at coarse resolutions (30 arc second grid) (e.g., [57, 58, 59]). It should also be noted that inferences from such coarse data are known to significantly overestimate cover estimates of marine organisms, potentially leading to incorrect interpretations by management agencies, such as amount of habitat contained in a particular area [60, 61]. Accordingly, if such data were not available at a similar resolution to that of the fine-scale MBS then we caution their use in such a modeling framework. However, if available at sufficient resolution, such variables should be considered as they will likely improve the precision and accuracy of epifaunal cover estimates, perhaps without the need for additional image scoring.

We also found that precision often remained low for morphospecies and associated parental CATAMI classes even if moderate accuracy was achieved. Understanding why an estimate of cover exhibits such low precision is clearly important. Perkins et al. [51] suggest that for organisms with < 10% cover, a characteristic commonly observed within AUV and other imagery, increased sample sizes may improve accuracy, while precision remains low. We suggest low precision could be a result of spatial, temporal, and residual variation. Spatial variance is the site-to-site variation, and may be reflective of the natural variability in the organism(s) of interest, and potentially not too much of a concern in the assessment of temporal fluctuations in organism(s). Temporal variance, however, is undesired because it can obscure trends over time. The effects of residual variation, which is due to seasonal variation during sampling, crew-to-crew differences in applying the monitoring protocol, and measurement error, can be reduced by means of a well-designed monitoring protocol and accurate survey tools (such as the AUV in the present study and in the future automated image processing). Importantly, however, it is only once a monitoring approach is implemented temporally, that the ability to disentangle these three sources of imprecision from one another can be achieved. This challenge remains to be addressed.

Finally, these results have important implications for scientists in how they report and communicate information to decision makers, and even to advance scientific understanding and improve future research. When survey results are reported without a clear description of their precision and accuracy, there is a risk that they will be over-interpreted by users. When results are presented as spatial areas on maps that can often appear to imply greater accuracy than is warranted from the data themselves. The communication of Type I and Type II errors have routinely been proposed as good practice in science to qualify research findings (e.g., [62]). Here we advocate for similar metrics are needed to communicate the accuracy and precision of inferences from distributional models (e.g., [61]). This research makes an important step in that direction, by identifying the constraints and limitations through a robust statistical approach to the design for monitoring programs and analysis of image data.

## Supporting information

S1 Fig An example of the morphometric variable complexity derived using the 3 m multibeam sonar data at the four analysis window scales. a) 3x3 (9 m), b) 9x9 (27 m), c) 17x17 (51 m), d) 33x33 (99 m), and e) 65x65 (195 m). Higher values mean more complex than lower values. Zoom boxes highlight the differences between analysis window scales.
(TIF)

S1 Table Description of variables used in models. * denotes those retained after correlation assessment.
(DOCX)

S2 Table List of morphospecies and parental CATAMI classes (hierarchical levels 1–6) observed using the autonomous underwater vehicle. (DOCX)

S3 Table Cover estimates (km^2^), and associated accuracy (explained deviance) and precision estimates (coefficients of variation) and for morphospecies and parental CATAMI classes (hierarchical levels 1–6). # denotes classes with too few observations for models converge.
(DOCX)
