# Supplementary figures and images for: An evaluation of the error and uncertainty in epibenthos cover estimates from AUV images collected with an efficient, spatially-balanced design
Source: PLoS One. 2018 Sep 18;13(9):e0203827. doi: 10.1371/journal.pone.0203827 (PMC6143229; doi:10.1371/journal.pone.0203827)

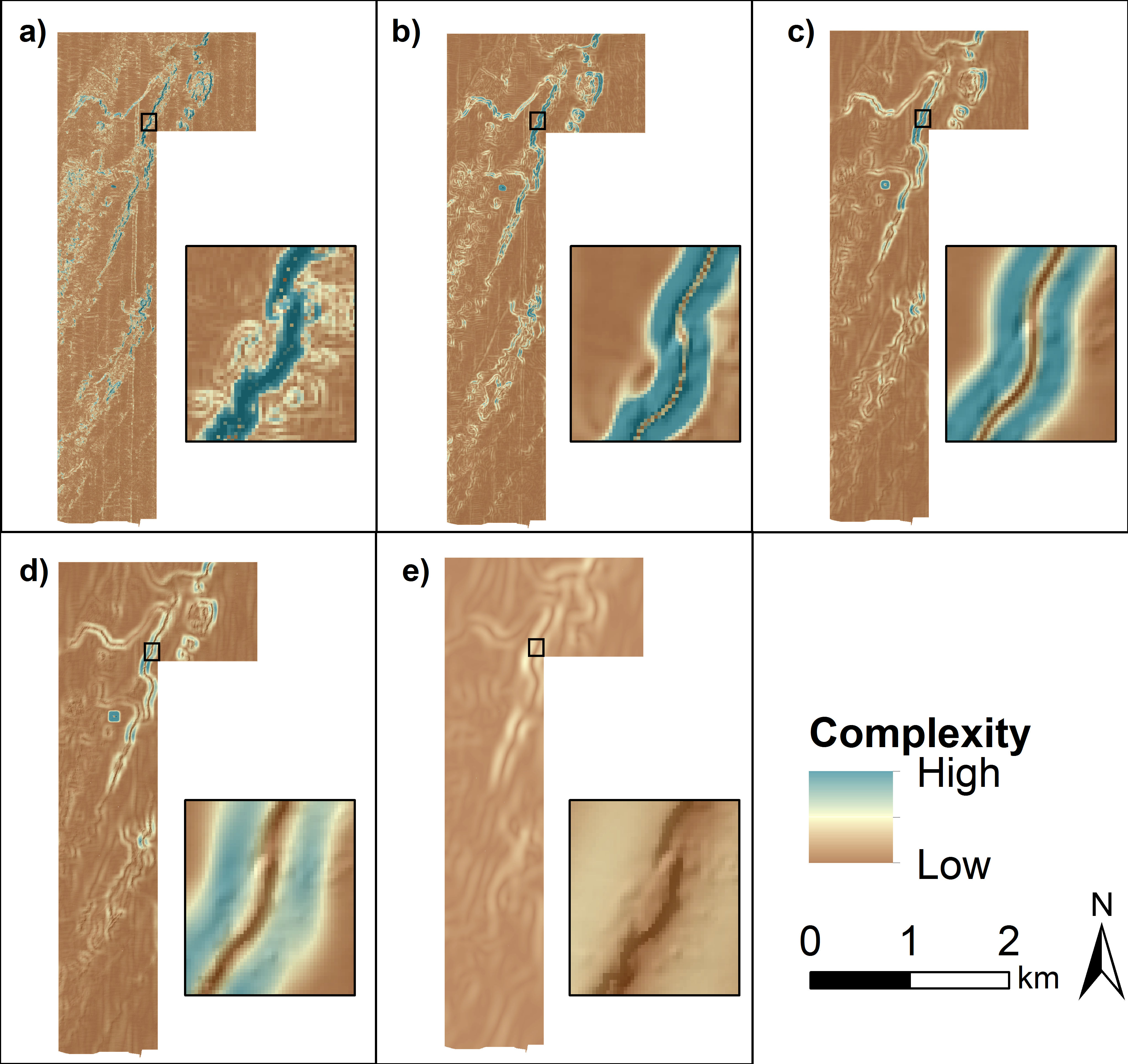

Supplement: S1 Fig — a) 3x3 (9 m), b) 9x9 (27 m), c) 17x17 (51 m), d) 33x33 (99 m), and e) 65x65 (195 m). Higher values mean more complex than lower values. Zoom boxes highlight the differences between analysis window scales. (TIF) [file pone.0203827.s001.tif]
