# Supplementary material for: An evaluation of the error and uncertainty in epibenthos cover estimates from AUV images collected with an efficient, spatially-balanced design
Source: PLoS One. 2018 Sep 18;13(9):e0203827. doi: 10.1371/journal.pone.0203827 (PMC6143229; doi:10.1371/journal.pone.0203827)
Supplement: S1 Table — * denotes those retained after correlation assessment. (DOCX) [file pone.0203827.s002.docx]

| **Variables** | **Variable description** | **Software** |
| --- | --- | --- |
| *Aspect*-  *Eastness (East)  *Northness (North) | Aspect is inherently circular, to overcome this, two trigonometric transformations [60] were applied; northness (sin(aspect)) and eastness (cos(aspect)). | Spatial Analyst- ArcGIS 10.1 |
| *Bathymetry (Bath) | Bathymetry provides a measure of water depth. | Caris |
| *Backscatter (Back) | Backscatter intensity is the acoustic scattering of the seafloor that represents the softness and hardness of the substratum. | Caris |
| *Complexity (Comp) | Complexity is the rate of change of the slope and is a measure of local variability in seabed. | LandSerf |
| *Latitude (Lat) | Latitudinal position of each cell in study area, calculated in WGS 1984 UTM 55s. | Spatial Analyst- ArcGIS 10.1 |
| *Longitude (Lon) | Longitudinal position of each cell in study area, calculated in WGS 1984 UTM 55s. | Spatial Analyst- ArcGIS 10.1 |
| *Maximum Curvature (Mcurv) | Maximum Curvature provides the greatest curve of either the profile or plan convexity relative to the analysis window [61]. | LandSerf |
| Plan Curvature | Plan curvature is perpendicular to the direction of the maximum slope. Positive values indicate upwardly concave, while negative values suggest upwardly convex. | LandSerf |
| Profile Curvature | Profile curvature is parallel to the direction of the maximum slope. Positive values indicate sidewardly convex, while negative values suggest sidewardly concave. | LandSerf |
| Rugosity | Rugosity provides the ratio of surface area to planar area within the analysis window and is to represent a measure of structural complexity [62]. | LandSerf |
| Slope | Slope is the maximum change in elevation between each cell and cells in its analysis neighborhood. Calculated in degrees from horizontal. | LandSerf |
