# Supplementary material for: An evaluation of the error and uncertainty in epibenthos cover estimates from AUV images collected with an efficient, spatially-balanced design
Source: PLoS One. 2018 Sep 18;13(9):e0203827. doi: 10.1371/journal.pone.0203827 (PMC6143229; doi:10.1371/journal.pone.0203827)
Supplement: S2 Table — (DOCX) [file pone.0203827.s003.docx]

| **CATAMI level** | **Class** | | | **Observations** | **No images with class present** |
| --- | --- | --- | --- | --- | --- |
| 1 | Ascidians | | | 13 | 12 |
| 1 | Bryozoans | | | 556 | 146 |
| 1 | Cnidarians | | | 30 | 28 |
| 1 | Fishes | | | 5 | 4 |
| 1 | Molluscs | | | 4 | 4 |
| 1 | Porifera | | | 258 | 179 |
| 1 | Worms | | | 2 | 1 |
| 2 | Ascidians (Unstalked) | | | 13 | 12 |
| 2 | Bryozoans (Hard) | | | 8 | 5 |
| 2 | Bryozoans (Soft) | | | 250 | 146 |
| 2 | Cnidarians (Colonial anemone) | | | 1 | 1 |
| 2 | Cnidarians (Corals) | | | 20 | 18 |
| 2 | Cnidarians (Hydrocorals) | 3 | | | 2 |
| 2 | Cnidarians (Hydroids) | | | 6 | 6 |
| 2 | Cnidarians (True anemone) | | | 3 | 3 |
| 2 | Fishes (Bony) | | | 2 | 2 |
| 2 | Fishes (Elasmobranchs) | | | 2 | 1 |
| 2 | Molluscs (Gastropods) | | | 4 | 4 |
| 2 | Porifera (Crusts) | | | 44 | 31 |
| 2 | Porifera (Erect) | | | 101 | 68 |
| 2 | Porifera (Hollow) | | | 28 | 22 |
| 2 | Porifera (Massive) | | | 85 | 58 |
| 2 | Worms (Polychaetes) | | | 2 | 1 |
| 2 | Worms (Sipuncula) | | 2 | | 2 |
| 3 | Ascidians (Unstalked Colonial) | | | 7 | 6 |
| 3 | Ascidians (Unstalked Solitary) | | | 6 | 6 |
| 3 | Bryozoans (Hard Fenestrate) | | | 8 | 5 |
| 3 | Bryozoans (Soft Foliose) | | | 24 | 14 |
| 3 | Cnidarians (Colonial anemone Zoanthids) | | | 1 | 1 |
| 3 | Cnidarians (Corals Black) | | | 18 | 16 |
| 3 | Cnidarians (Corals Stony) | | | 2 | 2 |
| 3 | Cnidarians (Other anemone) | | | 3 | 3 |
| 3 | Porifera (Crusts Creeping) | | | 31 | 20 |
| 3 | Porifera (Crusts Encrusting) | | | 13 | 11 |
| 3 | Porifera (Erect Branching) | | | 75 | 51 |
| 3 | Porifera (Erect Laminar) | | | 13 | 7 |
| 3 | Porifera (Erect Palmate) | | | 1 | 1 |
| 3 | Porifera (Hollow Cup like) | | | 19 | 13 |
| 3 | Porifera (Hollow Tubes and Chimneys) | | | 7 | 7 |
| 3 | Porifera (Massive Ball) | | | 3 | 3 |
| 3 | Porifera (Massive Barrels) | | | 2 | 2 |
| 3 | Porifera (Massive Cryptic) | | | 3 | 3 |
| 3 | Porifera (Massive Simple) | | | 91 | 61 |
| 3 | Worms (Polychaetes Tube worms) | | | 2 | 1 |
| 4 | Cnidarians (Corals Black Bramble) | | | 6 | 5 |
| 4 | Cnidarians (Corals Black Fans) | | | 6 | 5 |
| 4 | Cnidarians (Corals Black Whips) | | | 6 | 6 |
| 4 | Cnidarians (Corals Stony Solitary/mushroom) | | | 2 | 2 |
| 4 | Porifera (Hollow Cuplike Curled) | | | 5 | 5 |
| 4 | Porifera (Hollow Cuplike Goblet) | | | 14 | 8 |
| 5 | Cnidarians (Corals Black Bramble Fleshy) | | | 1 | 1 |
| 5 | Cnidarians (Corals Black Bramble non Fleshy) | | | 5 | 4 |
| 5 | Cnidarians (Corals Black Fans fern-frond) | | | 6 | 5 |
| 5 | Cnidarians (Corals Stony Solitary/mushroom solitary) | | | 2 | 2 |
| 6 | Cnidarians (Corals Black Bramble Fleshy Arborescent) | | | 1 | 1 |
| 6 | Cnidarians (Corals Black Bramble non Fleshy Arborescent) | | | 5 | 4 |
| 6 | Cnidarians (Corals Black Fans fern-frond complex) | | | 6 | 5 |
| Morphospecies | Ascidians (Ascidian 10 colonial purple) | | | 2 | 1 |
| Morphospecies | Ascidians (Ascidian 11 colonial) | | | 2 | 2 |
| Morphospecies | Ascidians (Ascidian 12 colonial Red) | | | 1 | 1 |
| Morphospecies | Ascidians (Ascidian 2 *Clavelina* like) | | | 1 | 1 |
| Morphospecies | Ascidians (Ascidian 6 Red throated) | | | 1 | 1 |
| Morphospecies | Ascidians (Ascidian 9 colonial) | | | 1 | 1 |
| Morphospecies | Ascidians (Ascidian Red Throated) | | | 1 | 1 |
| Morphospecies | Ascidians (Ascidian Unknown Solitary) | | | 4 | 4 |
| Morphospecies | Biota (Unknown Biology) | | | 1 | 1 |
| Morphospecies | Bryozoa (Bryozoa Hard Sparse) | | | 3 | 1 |
| Morphospecies | Bryozoa (Bryozoan 1 *Steginoprella* like) | | | 1 | 1 |
| Morphospecies | Bryozoa (Bryozoan 3 *Cantinicella* like) | | | 17 | 9 |
| Morphospecies | Bryozoa (Bryozoan 4 hard *Celleporaria* like) | | | 1 | 1 |
| Morphospecies | Bryozoa (Bryozoan 5 Lace) | | | 1 | 1 |
| Morphospecies | Bryozoa (Bryozoan 6 dark red) | | | 1 | 1 |
| Morphospecies | Bryozoa (Bryozoan 7 Hard) | | | 2 | 1 |
| Morphospecies | Bryozoa (Bryozoan Unknown Soft) | | | 6 | 4 |
| Morphospecies | Cnidaria (Anemone sp1) | | | 3 | 3 |
| Morphospecies | Cnidaria (bramble *Acabaria* sp) | | | 4 | 3 |
| Morphospecies | Cnidaria (bramble *Asperaxis kareni*) | | | 1 | 1 |
| Morphospecies | Cnidaria (Coral orange solitary) | | | 2 | 2 |
| Morphospecies | Cnidaria (Gorgonian pink 1) | | | 1 | 1 |
| Morphospecies | Cnidaria (Gorgonian red 2) | | | 5 | 4 |
| Morphospecies | Cnidaria (Hydroid Orange 2D) | | | 1 | 1 |
| Morphospecies | Cnidaria (Hydroid 1) | | | 1 | 1 |
| Morphospecies | Cnidaria (Hydroid 2) | | | 1 | 1 |
| Morphospecies | Cnidaria (Hydroid Brown Feathers) | | | 2 | 2 |
| Morphospecies | Cnidaria (Hydroid White) | | | 1 | 1 |
| Morphospecies | Cnidaria (Sea whip 1) | | | 6 | 6 |
| Morphospecies | Cnidaria (Soft coral 3 dark red) | | | 1 | 1 |
| Morphospecies | Cnidaria (Zoanthids 1 cf Epizoanthus) | | | 1 | 1 |
| Morphospecies | Cnidaria/Bryozoa/Hydroid matrix | | | 524 | 127 |
| Morphospecies | Fishes (*Caesioperca lepidoptera*) | | | 1 | 1 |
| Morphospecies | Fishes (Unknown Fish) | | | 1 | 1 |
| Morphospecies | Fishes (Unknown Teleost) | | | 1 | 1 |
| Morphospecies | Fishes (*Urolophus paucimaculatus*) | | | 2 | 1 |
| Morphospecies | Molluscs (Spindle Shell) | | | 1 | 1 |
| Morphospecies | Molluscs (Volute) | | | 3 | 3 |
| Morphospecies | Porifera (Arborescent 10 orange/brown fingers) | | | 2 | 2 |
| Morphospecies | Porifera (Arborescent 13 orange) | | | 1 | 1 |
| Morphospecies | Porifera (Arborescent 17 stumpy grey) | | | 13 | 8 |
| Morphospecies | Porifera (Arborescent 2 grey) | | | 1 | 1 |
| Morphospecies | Porifera (Arborescent 3 purple thin) | | | 2 | 1 |
| Morphospecies | Porifera (Arborescent 6 yellow) | | | 5 | 1 |
| Morphospecies | Porifera (Arborescent 8 tan) | | | 2 | 2 |
| Morphospecies | Porifera (Arborescent 9 orange thin) | | | 6 | 5 |
| Morphospecies | Porifera (Barrel Red Thick Wall) | | | 1 | 1 |
| Morphospecies | Porifera (Branching 1 Orange) | | | 2 | 2 |
| Morphospecies | Porifera (Branching 2 Brown) | | | 8 | 6 |
| Morphospecies | Porifera (Branching 3 Purple) | | | 3 | 1 |
| Morphospecies | Porifera (Branching 4 Brown) | | | 1 | 1 |
| Morphospecies | Porifera (Branching Beige Spindles) | | | 12 | 9 |
| Morphospecies | Porifera (Branching Beige Stumpy) | | | 1 | 1 |
| Morphospecies | Porifera (Branching Grey Fine Repent Like) | | | 2 | 1 |
| Morphospecies | Porifera (Branching Grey Repent Like) | | | 1 | 1 |
| Morphospecies | Porifera (Branching Grey Thorny) | | | 1 | 1 |
| Morphospecies | Porifera (Branching Orange Frilly ) | | | 3 | 2 |
| Morphospecies | Porifera (Branching Orange Long Fine) | | | 5 | 1 |
| Morphospecies | Porifera (Branching Purple Ramose Like) | | | 2 | 2 |
| Morphospecies | Porifera (Branching White Pointed) | | | 1 | 1 |
| Morphospecies | Porifera (Chimney Grey Single) | | | 3 | 3 |
| Morphospecies | Porifera (Cryptic 1 red) | | | 3 | 3 |
| Morphospecies | Porifera (Cup 7 light pink flat thick) | | | 1 | 1 |
| Morphospecies | Porifera (Cup 8 yellow) | | | 7 | 4 |
| Morphospecies | Porifera (Cup Red Smooth) | | | 1 | 1 |
| Morphospecies | Porifera (Cup Stalked Purple) | | | 5 | 2 |
| Morphospecies | Porifera (Encrusting 1 orange) | | | 2 | 2 |
| Morphospecies | Porifera (Encrusting 4 blue) | | | 1 | 1 |
| Morphospecies | Porifera (Encrusting 6 white) | | | 2 | 1 |
| Morphospecies | Porifera (Encrusting Beige Oscula) | | | 1 | 1 |
| Morphospecies | Porifera (Encrusting Black Lumpy) | | | 1 | 1 |
| Morphospecies | Porifera (Encrusting Purple Lumpy) | | | 1 | 1 |
| Morphospecies | Porifera (Encrusting White Granular) | | | 1 | 1 |
| Morphospecies | Porifera (Encrusting White Lumpy) | | | 3 | 2 |
| Morphospecies | Porifera (Encrusting Yellow Thick) | | | 1 | 1 |
| Morphospecies | Porifera (Fan 11 thick pink) | | | 1 | 1 |
| Morphospecies | Porifera (Fan 12 brown thin) | | | 1 | 1 |
| Morphospecies | Porifera (Fan 13 orange frilly) | | | 2 | 2 |
| Morphospecies | Porifera (Fan 4 pink) | | | 3 | 3 |
| Morphospecies | Porifera (Fan 9 orange thick) | | | 6 | 2 |
| Morphospecies | Porifera (Fan White Thick) | | | 1 | 1 |
| Morphospecies | Porifera (Globular 5 grey) | | | 1 | 1 |
| Morphospecies | Porifera (Laminar Grey Fungi) | | | 2 | 1 |
| Morphospecies | Porifera (Laminar White Small) | | | 2 | 1 |
| Morphospecies | Porifera (Lumpy 2 orange) | | | 6 | 4 |
| Morphospecies | Porifera (Lumpy 5 Yellow) | | | 25 | 10 |
| Morphospecies | Porifera (Lumpy 6 opaque yellow) | | | 1 | 1 |
| Morphospecies | Porifera (Lumpy Shapeless Grey) | | | 2 | 2 |
| Morphospecies | Porifera (Massive 18 orange holey) | | | 1 | 1 |
| Morphospecies | Porifera (Massive 19 yellow shapeless) | | | 2 | 2 |
| Morphospecies | Porifera (Massive 20 pink) | | | 2 | 1 |
| Morphospecies | Porifera (Massive 21) | | | 1 | 1 |
| Morphospecies | Porifera (Massive 22 Yellow holey) | | | 2 | 2 |
| Morphospecies | Porifera (Massive 23 Orange Ribbon) | | | 4 | 3 |
| Morphospecies | Porifera (Massive 24 Blue Lumpy) | | | 1 | 1 |
| Morphospecies | Porifera (Massive 3 orange) | | | 1 | 1 |
| Morphospecies | Porifera (Massive 4 donut) | | | 1 | 1 |
| Morphospecies | Porifera (Massive Beige Shapeless) | | | 2 | 2 |
| Morphospecies | Porifera (Massive Grey Laminar Like) | | | 4 | 1 |
| Morphospecies | Porifera (Massive Peach Shapeless Oscula) | | | 2 | 1 |
| Morphospecies | Porifera (Massive Yellow Irregular Ball) | | | 2 | 2 |
| Morphospecies | Porifera (Orange Massive Ball 1) | | | 1 | 1 |
| Morphospecies | Porifera (Palmate Grey Fingers) | | | 1 | 1 |
| Morphospecies | Porifera (Papillate 5 Black Ball) | | | 2 | 2 |
| Morphospecies | Porifera (Ramose Single Cream) | | | 8 | 6 |
| Morphospecies | Porifera (Repent 1 brown) | | | 1 | 1 |
| Morphospecies | Porifera (Repent 2 brown) | | | 20 | 11 |
| Morphospecies | Porifera (Simple Beige Irregular Oscula) | | | 4 | 3 |
| Morphospecies | Porifera (Simple Beige Laminar Like) | | | 1 | 1 |
| Morphospecies | Porifera (Simple Blue Shapeless) | | | 1 | 1 |
| Morphospecies | Porifera (Simple erect 1 cream) | | | 11 | 8 |
| Morphospecies | Porifera (Simple erect 2 Pink) | | | 1 | 1 |
| Morphospecies | Porifera (Simple Grey Creep) | | | 1 | 1 |
| Morphospecies | Porifera (Simple Grey Doughnut) | | | 4 | 3 |
| Morphospecies | Porifera (Simple Orange Confused) | | | 1 | 1 |
| Morphospecies | Porifera (Simple Orange Smooth) | | | 2 | 2 |
| Morphospecies | Porifera (Simple Purple Furrowed) | | | 1 | 1 |
| Morphospecies | Porifera (Simple Purple Shapeless) | | | 1 | 1 |
| Morphospecies | Porifera (Simple Red Ball Like) | | | 1 | 1 |
| Morphospecies | Porifera (Simple Red Globes) | | | 2 | 1 |
| Morphospecies | Porifera (Simple Yellow Lumpy) | | | 2 | 1 |
| Morphospecies | Porifera (Tube Beige Irregular) | | | 1 | 1 |
| Morphospecies | Porifera (Tubes Beige Prostrate) | | | 2 | 2 |
| Morphospecies | Porifera (Tubular 15 Fuzzy) | | | 1 | 1 |
| Morphospecies | Porifera (Yellow French Fires 1) | | | 1 | 1 |
| Morphospecies | Porifera (Yellow Shapeless Smooth 1) | | | 1 | 1 |
| Morphospecies | Worms (Tube Worm sp1) | | | 2 | 1 |
