# Supplementary material for: An evaluation of the error and uncertainty in epibenthos cover estimates from AUV images collected with an efficient, spatially-balanced design
Source: PLoS One. 2018 Sep 18;13(9):e0203827. doi: 10.1371/journal.pone.0203827 (PMC6143229; doi:10.1371/journal.pone.0203827)
Supplement: S3 Table — # denotes classes with too few observations for models converge. (DOCX) [file pone.0203827.s004.docx]

| **CATAMI level** | **Class** | **Area (km^2^) (%)** | **Precision (C.V.)** | **Accuracy (d^2^)** |
| --- | --- | --- | --- | --- |
| 1 | Ascidians | 0.006 (0.11) | 0.28 | 8.0 |
| 1 | Bryozoans | 0.285 (4.75) | 0.04 | 73.2 |
| 1 | Cnidarians | 0.028 (0.46) | 0.29 | 49.4 |
| 1 | Fishes | 0.002 (0.04) | 0.45 | 17.0 |
| 1 | Molluscs | # |  |  |
| 1 | Porifera | 0.42 (7) | 0.30 | 79.8 |
| 1 | Worms | # |  |  |
| 2 | Ascidians (Unstalked) | 0.006 (0.11) | 0.28 | 8.0 |
| 2 | Bryozoans (Hard) | 0.015 (0.25) | 0.24 | 10.9 |
| 2 | Bryozoans (Soft) | 0.302 (5.04) | 0.05 | 70.3 |
| 2 | Cnidarians (Colonial anemone) | # |  |  |
| 2 | Cnidarians (Corals) | 0.013 (0.22) | 0.26 | 8.4 |
| 2 | Cnidarians (Hydroids) | 0.003 (0.05) | 0.41 | 7.9 |
| 2 | Cnidarians (True anemone) | # |  |  |
| 2 | Fishes (Bony) | # |  |  |
| 2 | Fishes (Elasmobranchs) | # |  |  |
| 2 | Molluscs (Gastropods) | # |  |  |
| 2 | Porifera (Crusts) | 0.02 (0.34) | 0.17 | 48.3 |
| 2 | Porifera (Erect) | 0.09 (1.51) | 0.54 | 47.6 |
| 2 | Porifera (Hollow) | 0.062 (1.03) | 0.73 | 76.1 |
| 2 | Porifera (Massive) | 0.168 (2.8) | 0.72 | 80.1 |
| 2 | Worms (Polychaetes) | # |  |  |
| 3 | Ascidians (Unstalked Colonial) | 0.015 (0.25) | 0.72 | 4.1 |
| 3 | Ascidians (Unstalked Solitary) | 0.003 (0.05) | 0.42 | 6.3 |
| 3 | Bryozoans (Hard Fenestrate) | 0.015 (0.25) | 0.24 | 10.9 |
| 3 | Bryozoans (Soft Foliose) | 0.302 (5.04) | 0.05 | 30.3 |
| 3 | Cnidarians (Colonial anemone Zoanthids) | # |  |  |
| 3 | Cnidarians (Corals Black) | 0.008 (0.14) | 0.24 | 15.9 |
| 3 | Cnidarians (Corals Stony) | # |  |  |
| 3 | Cnidarians (Other anemone) | # |  |  |
| 3 | Porifera (Crusts Creeping) | 0.017 (0.28) | 0.20 | 49.2 |
| 3 | Porifera (Crusts Encrusting) | 0.007 (0.11) | 0.44 | 5.2 |
| 3 | Porifera (Erect Branching) | 0.054 (0.9) | 0.21 | 43.8 |
| 3 | Porifera (Erect Laminar) | 0.028 (0.46) | 0.98 | 48.1 |
| 3 | Porifera (Erect Palmate) | # |  |  |
| 3 | Porifera (Hollow Cup like) | 0.009 (0.15) | 0.24 | 29.9 |
| 3 | Porifera (Hollow Tubes and Chimneys) | 0.004 (0.06) | 0.38 | 6.2 |
| 3 | Porifera (Massive Ball) | # |  |  |
| 3 | Porifera (Massive Barrels) | # |  |  |
| 3 | Porifera (Massive Cryptic) | # |  |  |
| 3 | Porifera (Massive Simple) | 0.036 (0.59) | 0.38 | 51.1 |
| 3 | Worms (Polychaetes Tube worms) | # |  |  |
| 4 | Cnidarians (Corals Black Bramble) | 0.003 (0.05) | 0.42 | 22.6 |
| 4 | Cnidarians (Corals Black Fans) | 0.003 (0.04) | 0.41 | 31.2 |
| 4 | Cnidarians (Corals Black Whips) | 0.003 (0.06) | 0.43 | 4.1 |
| 4 | Cnidarians (Corals Stony Solitary/mushroom) | # |  |  |
| 4 | Porifera (Hollow Cuplike Curled) | 0.002 (0.04) | 0.45 | 18.6 |
| 4 | Porifera (Hollow Cuplike Goblet) | 0.007 (0.12) | 0.27 | 24.0 |
| 5 | Cnidarians (Corals Black Bramble Fleshy) | # |  |  |
| 5 | Cnidarians (Corals Black Bramble non Fleshy) | 0.003 (0.05) | 0.51 | 34.5 |
| 5 | Cnidarians (Corals Black Fans fern-frond) | 0.003 (0.04) | 0.41 | 31.2 |
| 5 | Cnidarians (Corals Stony Solitary/mushroom solitary) | # |  |  |
| 6 | Cnidarians (Corals Black Bramble Fleshy Arborescent) | # |  |  |
| 6 | Cnidarians (Corals Black Bramble non Fleshy Arborescent) | 0.003 (0.05) | 0.51 | 34.5 |
| 6 | Cnidarians (Corals Black Fans fern-frond complex ) | 0.003 (0.04) | 0.41 | 31.2 |
| Morphospecies | Ascidians (Ascidian 10 colonial purple) | # |  |  |
| Morphospecies | Ascidians (Ascidian 11 colonial) | # |  |  |
| Morphospecies | Ascidians (Ascidian 12 colonial Red) | # |  |  |
| Morphospecies | Ascidians (Ascidian 2 *Clavelina* like) | # |  |  |
| Morphospecies | Ascidians (Ascidian 6 Red throated) | # |  |  |
| Morphospecies | Ascidians (Ascidian 9 colonial) | # |  |  |
| Morphospecies | Ascidians (Ascidian Red Throated) | # |  |  |
| Morphospecies | Ascidians (Ascidian Unknown Solitary) | # |  |  |
| Morphospecies | Biota (Unknown Biology) | # |  |  |
| Morphospecies | Bryozoa (Bryozoa Hard Sparse) | # |  |  |
| Morphospecies | Bryozoa (Bryozoan 1 *Steginoprella* like) | # |  |  |
| Morphospecies | Bryozoa (Bryozoan 3 *Cantinicella* like) | 0.001 (0.01) | 0.57 | 67.6 |
| Morphospecies | Bryozoa (Bryozoan 4 hard *Celleporaria* like) | # |  |  |
| Morphospecies | Bryozoa (Bryozoan 5 Lace) | # |  |  |
| Morphospecies | Bryozoa (Bryozoan 6 dark red) | # |  |  |
| Morphospecies | Bryozoa (Bryozoan 7 Hard) | # |  |  |
| Morphospecies | Bryozoa (Bryozoan Unknown Soft) | 0.003 (0.05) | 0.34 | 5.06 |
| Morphospecies | Cnidaria (Anemone sp1) | # |  |  |
| Morphospecies | Cnidaria (bramble *Acabaria* sp) | # |  |  |
| Morphospecies | Cnidaria (bramble *Asperaxis kareni*) | # |  |  |
| Morphospecies | Cnidaria (Coral orange solitary) | # |  |  |
| Morphospecies | Cnidaria (Gorgonian pink 1) | # |  |  |
| Morphospecies | Cnidaria (Gorgonian red 2) | 0.004 (0.06) | 0.62 | 14.6 |
| Morphospecies | Cnidaria (Hydroid Orange 2D) | # |  |  |
| Morphospecies | Cnidaria (Hydroid 1) | # |  |  |
| Morphospecies | Cnidaria (Hydroid 2) | # |  |  |
| Morphospecies | Cnidaria (Hydroid Brown Feathers) | # |  |  |
| Morphospecies | Cnidaria (Hydroid White) | # |  |  |
| Morphospecies | Cnidaria (Sea whip 1) | 0.003 (0.06) | 0.43 | 4.05 |
| Morphospecies | Cnidaria (Soft coral 3 dark red) | # |  |  |
| Morphospecies | Cnidaria (Zoanthids 1 cf Epizoanthus) | # |  |  |
| Morphospecies | Cnidaria/Bryozoa/Hydroid matrix | 0.318 (5.3) | 0.06 | 58 |
| Morphospecies | Fishes (*Caesioperca lepidoptera*) | # |  |  |
| Morphospecies | Fishes (Unknown Fish) | # |  |  |
| Morphospecies | Fishes (Unknown Teleost) | # |  |  |
| Morphospecies | Fishes (*Urolophus paucimaculatus*) | # |  |  |
| Morphospecies | Molluscs (Spindle Shell) | # |  |  |
| Morphospecies | Molluscs (Volute) | # |  |  |
| Morphospecies | Porifera (Arborescent 10 orange/brown fingers) | # |  |  |
| Morphospecies | Porifera (Arborescent 13 orange) | # |  |  |
| Morphospecies | Porifera (Arborescent 17 stumpy grey) | 0.0003 (0.005) | 0.46 | 55.4 |
| Morphospecies | Porifera (Arborescent 2 grey) | # |  |  |
| Morphospecies | Porifera (Arborescent 3 purple thin) | # |  |  |
| Morphospecies | Porifera (Arborescent 6 yellow) | 0.004 (0.06) | 0.68 | 6.49 |
| Morphospecies | Porifera (Arborescent 8 tan) | # |  |  |
| Morphospecies | Porifera (Arborescent 9 orange thin) | 0.003 (0.05) | 0.41 | 28.6 |
| Morphospecies | Porifera (Barrel Red Thick Wall) | # |  |  |
| Morphospecies | Porifera (Branching 1 Orange) | # |  |  |
| Morphospecies | Porifera (Branching 2 Brown) | 0.004 (0.06) | 0.35 | 20.8 |
| Morphospecies | Porifera (Branching 3 Purple) | # |  |  |
| Morphospecies | Porifera (Branching 4 Brown) | # |  |  |
| Morphospecies | Porifera (Branching Beige Spindles) | 0.005 (0.08) | 0.30 | 14 |
| Morphospecies | Porifera (Branching Beige Stumpy) | # |  |  |
| Morphospecies | Porifera (Branching Grey Fine Repent Like) | # |  |  |
| Morphospecies | Porifera (Branching Grey Repent Like) | # |  |  |
| Morphospecies | Porifera (Branching Grey Thorny) | # |  |  |
| Morphospecies | Porifera (Branching Orange Frilly ) | # |  |  |
| Morphospecies | Porifera (Branching Orange Long Fine) | 0.017 (0.28) | 0.67 | 7.26 |
| Morphospecies | Porifera (Branching Purple Remose Like) | # |  |  |
| Morphospecies | Porifera (Branching White Pointed) | # |  |  |
| Morphospecies | Porifera (Chimney Grey Single) | # |  |  |
| Morphospecies | Porifera (Cryptic 1 red) | # |  |  |
| Morphospecies | Porifera (Cup 7 light pink flat thick) | # |  |  |
| Morphospecies | Porifera (Cup 8 yellow) | 0.004 (0.06) | 0.41 | 24.1 |
| Morphospecies | Porifera (Cup Red Smooth) | # |  |  |
| Morphospecies | Porifera (Cup Stalked Purple) | 0.003 (0.05) | 0.50 | 6.08 |
| Morphospecies | Porifera (Encrusting 1 orange) | # |  |  |
| Morphospecies | Porifera (Encrusting 4 blue) | # |  |  |
| Morphospecies | Porifera (Encrusting 6 white) | # |  |  |
| Morphospecies | Porifera (Encrusting Beige Oscula) | # |  |  |
| Morphospecies | Porifera (Encrusting Black Lumpy) | # |  |  |
| Morphospecies | Porifera (Encrusting Purple Lumpy) | # |  |  |
| Morphospecies | Porifera (Encrusting White Granular) | # |  |  |
| Morphospecies | Porifera (Encrusting White Lumpy) | # |  |  |
| Morphospecies | Porifera (Encrusting Yellow Thick) | # |  |  |
| Morphospecies | Porifera (Fan 11 thick pink) | # |  |  |
| Morphospecies | Porifera (Fan 12 brown thin) | # |  |  |
| Morphospecies | Porifera (Fan 13 orange frilly) | # |  |  |
| Morphospecies | Porifera (Fan 4 pink) | # |  |  |
| Morphospecies | Porifera (Fan 9 orange thick) | 0.003 (0.04) | 0.42 | 35.4 |
| Morphospecies | Porifera (Fan White Thick) | # |  |  |
| Morphospecies | Porifera (Globular 5 grey) | # |  |  |
| Morphospecies | Porifera (Laminar Grey Fungi) | # |  |  |
| Morphospecies | Porifera (Laminar White Small) | # |  |  |
| Morphospecies | Porifera (Lumpy 2 orange) | 0.003 (0.05) | 0.84 | 12.6 |
| Morphospecies | Porifera (Lumpy 5 Yellow) | 0.012 (0.2) | 0.21 | 33.7 |
| Morphospecies | Porifera (Lumpy 6 opaque yellow) | # |  |  |
| Morphospecies | Porifera (Lumpy Shapeless Grey) | # |  |  |
| Morphospecies | Porifera (Massive 18 orange holey) | # |  |  |
| Morphospecies | Porifera (Massive 19 yellow shapeless) | # |  |  |
| Morphospecies | Porifera (Massive 20 pink) | # |  |  |
| Morphospecies | Porifera (Massive 21) | # |  |  |
| Morphospecies | Porifera (Massive 22 Yellow holey) | # |  |  |
| Morphospecies | Porifera (Massive 23 Orange Ribbon) | # |  |  |
| Morphospecies | Porifera (Massive 24 Blue Lumpy) | # |  |  |
| Morphospecies | Porifera (Massive 3 orange) | # |  |  |
| Morphospecies | Porifera (Massive 4 donut) | # |  |  |
| Morphospecies | Porifera (Massive Beige Shapeless) | # |  |  |
| Morphospecies | Porifera (Massive Grey Laminar Like) | # |  |  |
| Morphospecies | Porifera (Massive Peach Shapeless Oscula) | # |  |  |
| Morphospecies | Porifera (Massive Yellow Irregular Ball ) | # |  |  |
| Morphospecies | Porifera (Orange Massive Ball 1) | # |  |  |
| Morphospecies | Porifera (Palmate Grey Fingers) | # |  |  |
| Morphospecies | Porifera (Papillate 5 Black Ball) | # |  |  |
| Morphospecies | Porifera (Remose Single Cream) | 0.004 (0.07) | 0.42 | 43 |
| Morphospecies | Porifera (Repent 1 brown) | # |  |  |
| Morphospecies | Porifera (Repent 2 brown) | 0.008 (0.14) | 0.25 | 19.2 |
| Morphospecies | Porifera (Simple Beige Irregular Oscula) | # |  |  |
| Morphospecies | Porifera (Simple Beige Laminar Like) | # |  |  |
| Morphospecies | Porifera (Simple Blue Shapeless) | # |  |  |
| Morphospecies | Porifera (Simple erect 1 cream) | 0.001 (0.01) | 0.10 | 24.9 |
| Morphospecies | Porifera (Simple erect 2 Pink) | # |  |  |
| Morphospecies | Porifera (Simple Grey Creep) | # |  |  |
| Morphospecies | Porifera (Simple Grey Doughnut ) | # |  |  |
| Morphospecies | Porifera (Simple Orange Confused) | # |  |  |
| Morphospecies | Porifera (Simple Orange Smooth) |  |  |  |
| Morphospecies | Porifera (Simple Purple Furrowed) | # |  |  |
| Morphospecies | Porifera (Simple Purple Shapeless) | # |  |  |
| Morphospecies | Porifera (Simple Red Ball Like) | # |  |  |
| Morphospecies | Porifera (Simple Red Globes ) | # |  |  |
| Morphospecies | Porifera (Simple Yellow Lumpy) | # |  |  |
| Morphospecies | Porifera (Tube Beige Irregular) | # |  |  |
| Morphospecies | Porifera (Tubes Beige Prostrate) | # |  |  |
| Morphospecies | Porifera (Tubular 15 Fuzzy) | # |  |  |
| Morphospecies | Porifera (Yellow French Fires 1) | # |  |  |
| Morphospecies | Porifera (Yellow Shapeless Smooth 1) | # |  |  |
| Morphospecies | Worms (Tube Worm sp1) | # |  |  |
